# Rebranding Inpatient Community Meetings

**DOI:** 10.1192/bjo.2025.10108

**Published:** 2025-06-20

**Authors:** Hannah Campling, Bharat Velani, Georgina Hearn

**Affiliations:** North London Foundation Trust, London, United Kingdom

## Abstract

**Aims:** The North London Foundation Trust was established in 2024. The partnership has created a new clinical strategy for the next 5 years (2024–2029) and some of the main priorities are: “For all services to use a trauma informed approach”, “Service users must be involved in co-production and decision making”, “Value feedback from service users”, “Facilitate communication and information sharing with service users”.

For these priorities to be addressed there need to be forums in which service users are able to be heard and fed back to using a trauma informed approach. Currently there are community meetings on the inpatient wards for service users and staff to feedback on any issues within the ward environment. On my ward these are poorly attended by both staff and patients and feedback from patients is that they raise the same issues, but nothing gets acted on. There is no set policy/protocol for how these meetings should run and who should be in attendance. By formalising the structure of the community meetings using a trauma informed framework, my hope is that both patients and staff benefit from the shared space and that the learning can be shared with other wards.

**Objectives:** To develop an evidence-based protocol for running community meetings on an inpatient psychiatric ward that fits within a trauma-informed framework; Improved attendance from staff and patients at the ward community meeting; Improved satisfaction from staff and patients attending community meetings; Share learning with other wards in the partnership.

**Methods:** A literature search to establish current best practice for running community meetings.

Qualitative questionnaires/ structured interviews and thematic analysis of staff interviews.

Development of protocol for running community meetings on inpatient wards.

3 month pilot of the new community meetings.

Attendance records pre and post intervention.

**Results:** Attendance records show improved attendance of both staff and patients at the weekly community meetings. Prior to the intervention, thematic analysis showed that staff thought there was a lack of clarity about goals, diverse interpretations of community meetings, and mixed expectations about patient involvement. Post intervention, analysis revealed that community meetings were widely appreciated as a valuable initiative that enhanced the ward culture, patient recovery, and staff-patient relationships. Despite challenges, many participants felt these meetings brought significant benefits.

**Conclusion:** Having a trauma-informed, semi-structured proforma for running inpatient community meetings helps to improve attendance, satisfaction and positive outcomes from the meetings for both staff and patients.

